# Topography-associated thermal gradient predicts warming effects on woody plant structural diversity in a subtropical forest

**DOI:** 10.1038/srep40387

**Published:** 2017-01-09

**Authors:** Siyan He, Yonglin Zhong, Yudan Sun, Zhiyao Su, Xiaorong Jia, Yanqiu Hu, Qing Zhou

**Affiliations:** 1 College of Forestry and Landscape Architecture, South China Agricultural University, Guangzhou 510642, China

## Abstract

Understanding global warming effects on forest ecosystems will help policy-makers and forest managers design forest management and biodiversity conservation strategies. We examined the change in woody plant structural diversity in response to topography-associated thermal gradients in a subtropical forest with diverse abundance patterns. We found that energy distribution in a warming trend across slopes had significant effects on woody plant structural diversity. Except for total basal area of the adult trees, plant structural diversity significantly decreased with the increase of heat load. Heat load is significantly and negatively correlated with number of stems, number of species, and the number of stems of the most abundant species (*N*_max_) for seedlings, saplings, and individuals of all sizes. For the adult trees, heat load is significantly and positively correlated with number of stems and *N*_max_, and negatively but not significantly with number of species, indicating that large trees may not be as sensitive as seedlings and saplings to warming. Partial correlation analysis, having controlled for elevation, strengthened those relations in most cases. Our results reveal that warming will increase community productivity by enhancing the growth of large trees, but decrease species diversity and inhibit the regeneration of tree seedlings and saplings.

Heterogeneity in energy distribution on forest habitats is regarded as one of the most important factors maintaining and driving plant structural diversity^1^,^2^, a type of diversity commonly measured as species diversity and size diversity, i.e., species richness, number of individuals, and tree size^3^,^4^. Higher habitat heterogeneity is associated with higher heterogeneity in energy distribution, which results in higher variation in woody plant structural diversity^5^. The driving factor of heterogeneous energy distribution varies on different scales. On a global scale, energy distribution is mainly controlled by broad-scale geographic gradient. With substantial energy decreasing from the equator poleward, plant diversity exhibits a latitudinal pattern along a thermal energy gradient^6^. However, on a local scale, especially at a forest stand level, thermal energy distribution is primarily affected by topography, resulting in spatial heterogeneity under different topographic conditions^5^,^7^. As a result, plant diversity patterns show considerable spatial heterogeneity in response to topography-associated energy distribution. Furthermore, variations in plant structural diversity patterns increase with a higher complexity of topographic conditions^5^,^8^.

As woody plant diversity patterns on small scales is potentially associated with topographic heterogeneity of energy distribution^9^, the influence of varying topography on energy distribution and woody plant structural diversity has attracted extensive attention from ecologists and biogeographers^10^,^11^,^12^,^13^. In the northern hemisphere, south and southwest slope aspects receive longer durations and greater intensities of solar radiation and consequently obtain more thermal energy as compared to north and northeast slope aspects. As a result, south and southwest slope aspects are warmer and drier than north and northeast slope aspects^7^. However, the relations of woody plant diversity patterns to the spatial heterogeneity of energy distribution were not consistent as reported by previous studies^11^,^13^,^14^. For example, a study in a montane temperate forest in Virginina, USA, indicated that the warmer southwest slope had higher species richness and stem density but lower basal area than the northeast slope^13^, while a study in an arid trans-Himalayan region of Nepal showed that woody plant diversity was lower on the warmer southwest slope than on northeast slope^14^. The discrepancy in these studies suggests that plant diversity patterns in response to aspect-related thermal gradient might be site-specific, and that factors such as moisture regimes and human disturbance played a role or interacted with the thermal effect. Therefore, to predict future warming scenario on woody plant structural diversity using the aspect-related thermal gradient as a “space-for-time” approach, we should select a site differing in aspects but homogeneous in other factors such as precipitation and human disturbance.

As a proxy variable of thermal energy, heat load is the heat gain from solar radiation, which reflects the theoretical thermal condition in a habitat^7^,^15^. The stand-level topography-associated heat load heterogeneity forms a moderate thermal gradient for observing changes in plant diversity patterns in response to warming, thus serving as a reference for predicting future plant diversity changes under global warming. In this study, we explored the effects of topography-associated heat load on woody plant structural diversity in a subtropical forest in south China. We aimed to address the following questions: (1) Is heat load significantly correlated with woody plant diversity, (2) How woody plant structural diversity attributes change across a thermal gradient, and (3) Do trees of different sizes differ in their response to warming?

## Methods

### Study area and sampling design

We conducted this study in the Kanghe Provincial Nature Reserve, which is located in the southeastern part of Guangdong province, China (23°44′–23°53′ N, 115°04′–115°09′E). The area lies within the subtropical monsoon climate zone, with a mean annual precipitation of 2,142.6 mm and a mean annual temperature ranging from 20.3–21.1 °C 16. The dominant vegetation in this area is subtropical broadleaved forest. A detailed description of the climate, geology, and floristic characteristics can be found in a previous study in this area by Hu *et al*.^16^.

**Figure 1:**
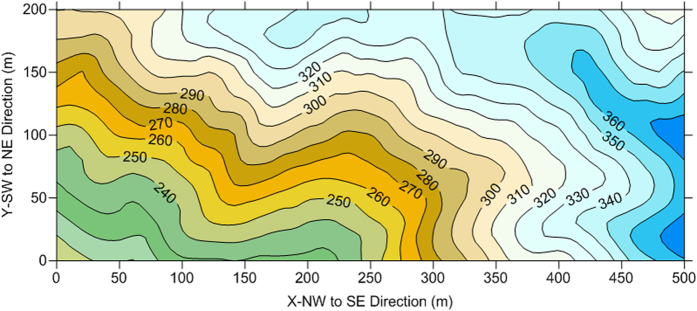
Topographic map of the Kanghe large plot. The topographic map is generated with the surveying data of the plot using the software Surfer 11.0 (Golden Software, Inc., Golden, Colorado, USA).

We established a 10-ha permanent plot within the nature reserve following the protocol for large forest census plots^17^. The permanent plot forms a rectangle of 500 m by 200 m, with the long axis running from northwest to southeast (Fig. 1). The 10-ha plot was further divided into 250 grid-cells or subplots (20 × 20 m) with surveying techniques using a total station (Nikon DTM-310), and each subplot was numbered and marked by placing PVC stakes at the corners. Grid-cell systems of 10 × 10 m and 5 × 5 m were also laid out using smaller PVC stakes for easy operation of plant census. Plot surveying was done by experts from the Surveying Department, South China Agricultural University.

### Plant census

We conducted a plant census in every 20 × 20 m subplot. All woody plants with a diameter at breast height (DBH, at 1.3 m above the ground) ≥ 1 cm were measured, identified, and tagged with unique numbers. All censused individuals were recorded with the species name and DBH, which was measured to the nearest 0.1 cm. Most individuals were identified to species on the spot. In cases where plant identity was uncertain, voucher specimens were collected and labelled for subsequent identification at the herbarium of South China Agricultural University (CANT). Plant systematics followed Ye and Peng^18^.

### Topographic data and heat load index

At the stage of plot surveying for the layout of grid-cell systems, the horizontal distance and the elevation at each corner of a subplot relative to the southwestern corner of the 10-ha plot, which was designated a value of 0, were recorded by the total station (Nikon DTM-310). These data became the source for the subsequent computation of average elevation, slope steepness, and slope aspect^19^, as well as the latitude-longitude coordinates of each subplot. The elevation of the 10-ha plot ranges from 200.0 to 379.6 m above sea level (a.s.l), and the average elevation of each 20 × 20 m plot from 209.1 to 371.9 m a.s.l. The slope of each subplot ranges from 7.9° to 45.8°, while aspect from 19.7° to 353.2°. In northern Hemisphere, solar radiation is greatest on southwest-facing slopes and least on northeast-facing slopes, thus resulting in an aspect-related gradient of heat or temperature^20^,^21^, because a southwest slope with the afternoon sun and thus longer duration of radiation in a day will have higher maximum temperature than a northeast slope with the morning sun^15^. Consequently, the subplots with various aspects were grouped into categories from a cold slope to a warmer slope with increasing thermal energy: shady aspect (northeast), semi-shady aspect (east, southeast, northwest, and north), semi-sunny aspect (south and west), and sunny aspect (southwest)^21^,^22^.

We computed estimates of heat load index for the subplots using the nonparametric multiplicative regression (NPMR) method following McCune^23^. To compute heat load index, we first constructed a data matrix with three predictor variables for each subplot, i.e., latitude, slope, and aspect. With a response matrix, a predictor matrix, and a model specification file provided by the author (http://people.oregonstate.edu/~mccuneb/radiation.htm), we used the predictor data matrix we constructed to generate predictions of heat load for new sites, i.e., points representing subplots in our study, using the software HyperNiche 2.0 (MjM Software, Gleneden Beach, Oregon, USA). The predicted heat load index was then used to correlate with structural diversity attributes in subsequent analyses.

### Statistical analysis

Two out of the 250 subplots surveyed, i.e., subplots 33 and 250, were excluded from analysis due to substantial outcrops of rugged granite there. Only 27 and 10 individuals from five and two species, which are not unique to the whole plot, occurred in subplots 33 and 250, respectively. Therefore, we constructed datasets comprising 248 subplots for all the subsequent analyses.

To test whether woody plants of different growth stages have different responses to warming, we grouped the recorded individuals into three size classes as defined by DBH for further analyses: Seedlings: 1 cm ≤ DBH ≤ 2.4 cm; Saplings: 2.5 cm ≤ DBH ≤ 12.4 cm; Adults: DBH ≥ 12.5 cm. Four structural diversity attributes for each subplot, i.e., number of stems, number of species, number of stems of the most abundant species (*N*_max_), and basal area, were calculated by adults, saplings, seedlings, and individuals of all sizes combined, respectively, using PC-ORD 6.0 (MjM Software, Gleneden Beach, Oregon, USA.). These attributes became the response variables in further analyses.

To visualize the diversity and dominance patterns by size class at plot level, we constructed rank abundance curves to show abundance distribution relative to species and the evenness within each size class. We also plotted bar charts to show the contrast between number of stems and total basal area by size class. These plot-level structural diversity patterns provided the basic information for later analyses across a topography-associated thermal energy gradient.

To relate the structural diversity attributes to heat load index, we calculated Pearson correlation coefficients for each size class between the plant structural diversity attributes and heat load index. However, difference in energy distribution may also due to changes in elevation, and there is an elevation difference of 162.8 m in our study plot. The effects of aspect-related and elevation-related energy gradients on plant structural diversity may be overlapped or offset. To avoid possible spurious correlations and to test the strengths of the relationships, we also performed partial correlation analysis, with elevation as the control variable. All the variables were log_10_-transformed to meet the assumptions before correlation and partial correlation analyses.

**Figure 2:**
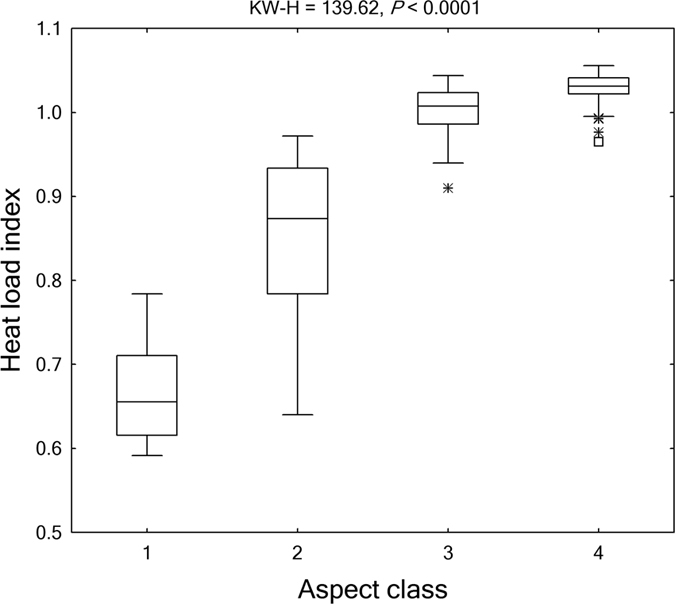
Heat load index across slope aspects. The horizontal line in each box is the median, the box endpoints represent the 25th and 75th percentile values, the whiskers represent the non-outlier range, and the asterisks and open squares indicate outliers and extremes, respectively. Differences across aspect classes were evaluated using Kruskal-Wallis H (KW-H) test. Aspect class: 1: shady aspect; 2: semi-shady aspect; 3: semi-sunny aspect; 4: sunny aspect.

We used Kruskal-Wallis test, a nonparametric statistical method, to test for significance of variation in the structural diversity attributes across the aspect-related thermal gradient. The thermal gradient from shady to sunny aspects represented a warming trend, which was verified by the significant change in heat load index across these aspect classes (Kruskal-Wallis test, *P* < 0.0001; Fig. 2). The Kruskal-Wallis tests were performed and contrasted by size classes, i.e., seedlings, saplings, adults, as well as individuals of all sizes combined.

Except for the prediction of heat load index and for the calculation of structural diversity attributes, all the other analyses were performed using Statistica 8.0 (Statsoft, Inc. Tulsa, OK, USA).

## Results

### Community-level structural diversity

**Figure 3:**
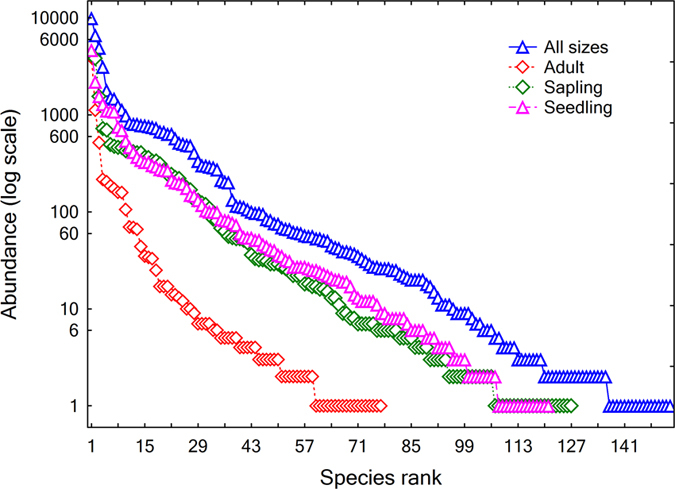
Species rank abundance curves showing species abundance distributions in the forest stand. Size class is classified for convenience. Seedling: 1 cm ≤ DBH ≤ 2.4 cm; Sapling: 2.5 cm ≤ DBH ≤ 12.4 cm; Adult: DBH ≥ 12.5 cm.

Within the 248 subplots, we recorded 50,032 stems of 153 woody plant species from 96 genera and 52 families (Supplementary Table S1). These species showed diverse abundance patterns. Only the five most abundant species together accounted for over 50% of the total number of stems, and the single most abundant species, *Castanopsis carlesii*, had 9,942 (19.9%) stems. In contrast, a total of 80 rare species (≤3 stems/ha), of which seventeen are singletons, were represented by only 637 (1.3%) individuals, although they constituted 52.3% of the total species richness. The rank abundance curves visually revealed such high dominance or rarity patterns (Fig. 3). The shapes of the rank abundance curves followed a log series distribution^24^ for seedlings, saplings, adult trees, and individuals of all sizes combined.

**Figure 4:**
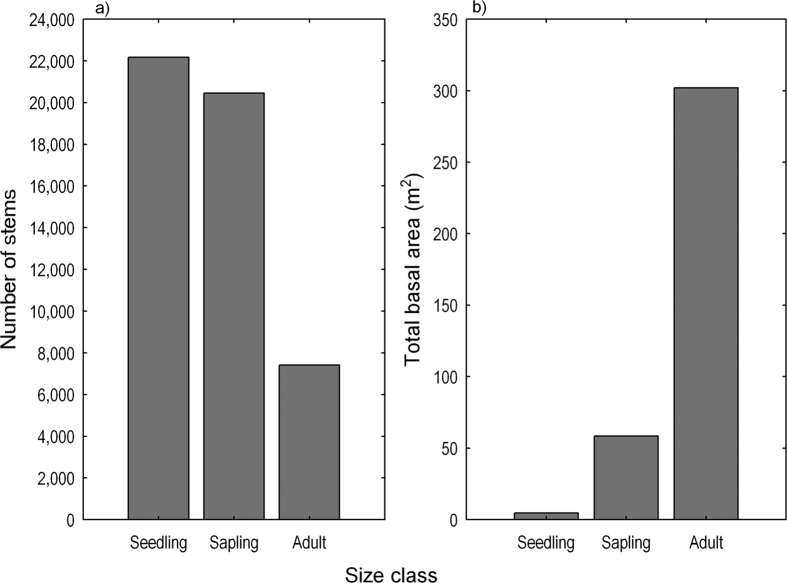
Bar charts showing number of stems (**a**) and total basal area (**b**) by size class. Size class is classified for convenience. Seedling: 1 cm ≤ DBH ≤ 2.4 cm; Sapling: 2.5 cm ≤ DBH ≤ 12.4 cm; Adult: DBH ≥ 12.5 cm.

The contrasting patterns were evident in total abundance and total basal area by size class (Fig. 4). The number of stems for seedlings, saplings, and adults were 22,173, 20,447, and 7,412 (Fig. 4a), accounting for 44.3%, 40.9%, and 14.8% of the total number of stems, respectively. However, with 44.3% of the total number of stems, seedlings together were responsible for only 1.3% (4.62 m^2^) of total basal area, while the adult trees, with only 14.8% of the total number of stems, contributed 301.96 m^2^ (82.7%) to the total basal area (Fig. 4b). In forest communities, tree basal area is a good surrogate variable for stand biomass or productivity, and is a predictor for modelling tree or stand volume. In view of the contrasting patterns, factors heavily influencing species diversity may have different effects on productivity, and vice versa.

### Correlations of heat load with plant structural diversity

Heat load index was highly significantly and negatively correlated with number of stems, number of species, and the number of stems of the most abundant species (*N*_max_) for seedlings, saplings, and individuals of all sizes combined. It was highly significantly and negatively with basal area for seedlings and saplings, and positively but not significantly with basal area for individuals of all sizes combined. For the adult trees, heat load index was significantly and positively correlated with number of stems, negatively but not significantly with number of species, positively but not significantly with *N*_max_, positively and highly significantly with basal area. Partial correlation analysis, after controlling for the elevation variable, strengthened those correlations in most cases (Table 1).

### Changes in structural diversity across an aspect-related thermal gradient

**Figure 5:**
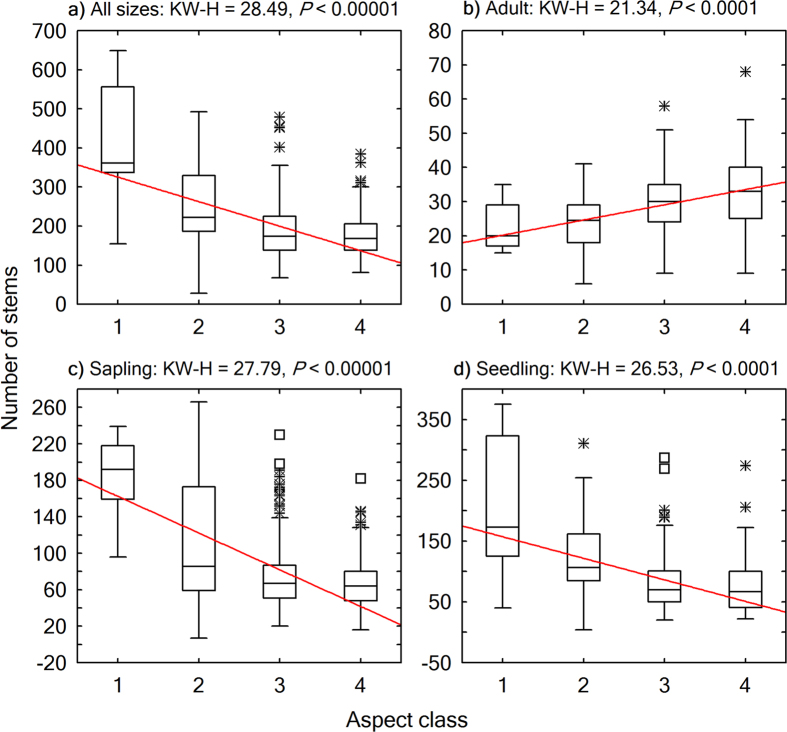
Boxplots showing variations of community-level total abundance across aspects. The horizontal line in each box is the median, the box endpoints represent the 25th and 75th percentile values, the whiskers represent the non-outlier range, and the asterisks and open squares indicate outliers and extremes, respectively. A linear fit for the medians was plotted to show the trend of change. Differences across aspect classes were evaluated using Kruskal-Wallis H (KW-H) test. Aspect class: 1: shady aspect; 2: semi-shady aspect; 3: semi-sunny aspect; 4: sunny aspect.

**Figure 6:**
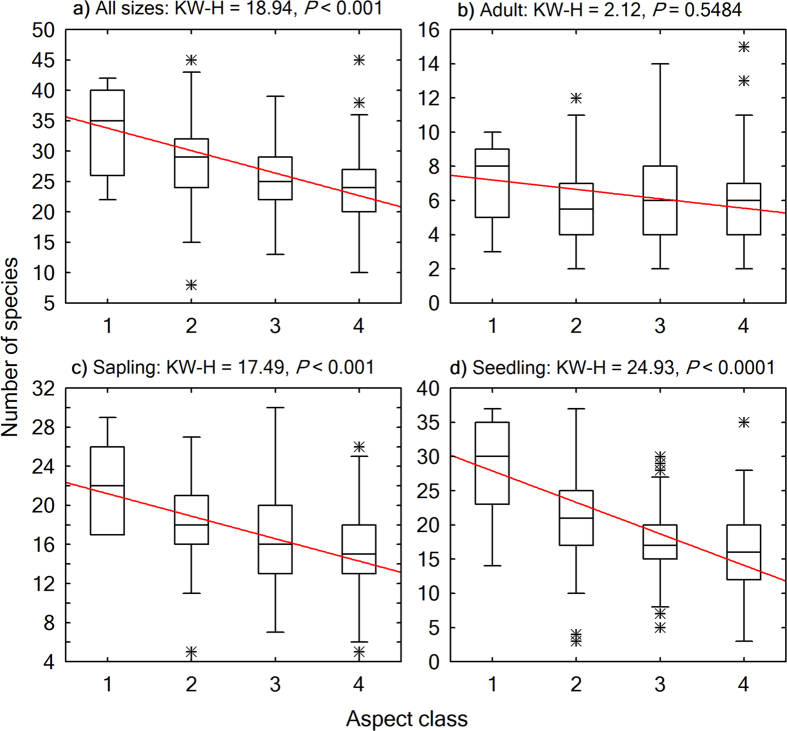
Boxplots showing variations of community-level species richness across aspects. The horizontal line in each box is the median, the box endpoints represent the 25th and 75th percentile values, the whiskers represent the non-outlier range, and the asterisks indicate outliers. A linear fit for the medians was plotted to show the trend of change. Differences across aspect classes were evaluated using Kruskal-Wallis H (KW-H) test. Aspect class: 1: shady aspect; 2: semi-shady aspect; 3: semi-sunny aspect; 4: sunny aspect.

**Figure 7:**
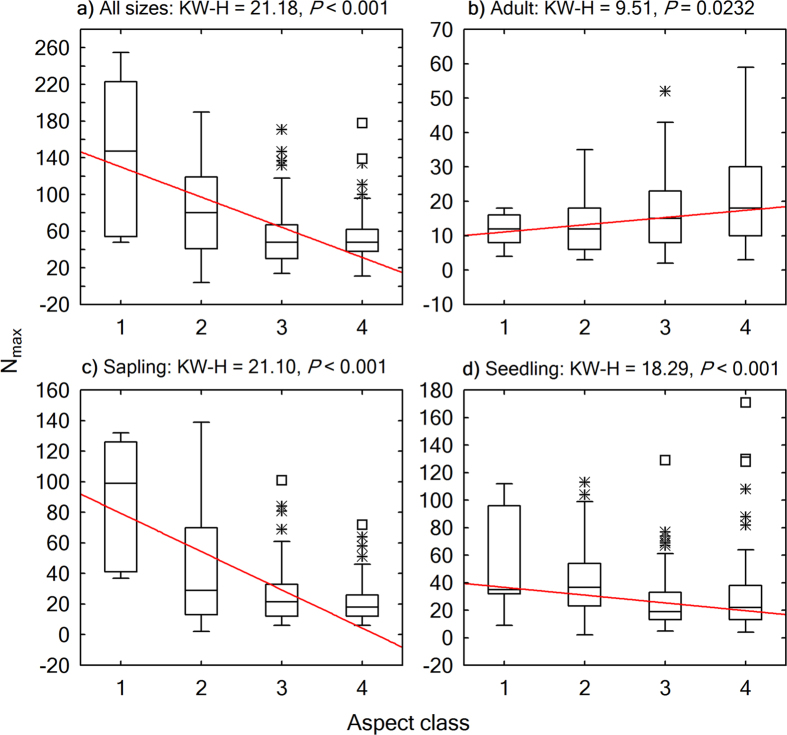
Boxplots showing variations in number of stems of the most-abundant species (*N*_max_) across aspects. The horizontal line in each box is the median, the box endpoints represent the 25th and 75th percentile values, the whiskers represent the non-outlier range, and the asterisks and open squares indicate outliers and extremes, respectively. A linear fit for the medians was plotted to show the trend of change. Differences across aspect classes were evaluated using Kruskal-Wallis H (KW-H) test. Aspect class: 1: shady aspect; 2: semi-shady aspect; 3: semi-sunny aspect; 4: sunny aspect.

**Figure 8:**
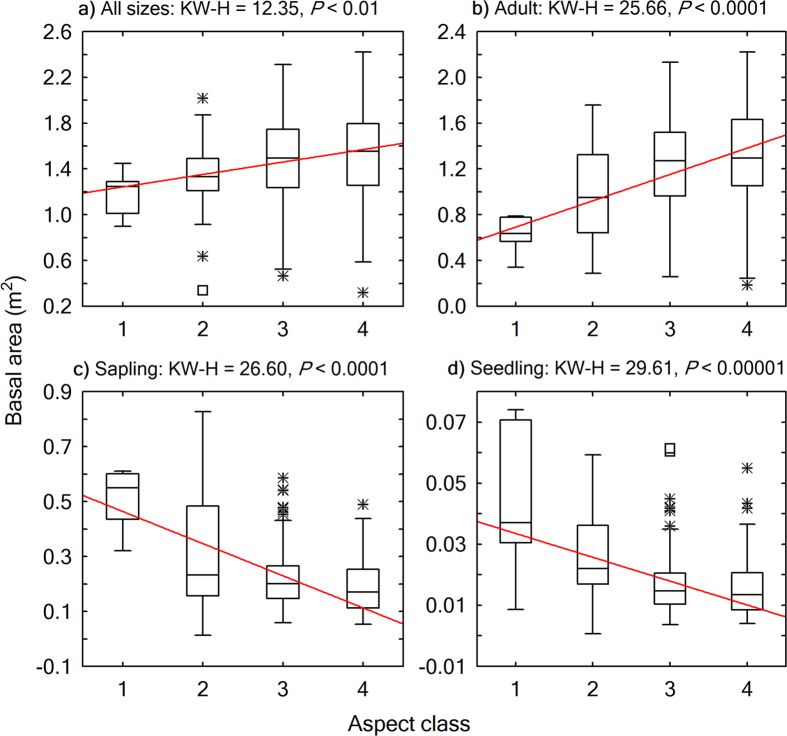
Boxplots showing variations of community-level total basal area across aspects. The horizontal line in each box is the median, the box endpoints represent the 25th and 75th percentile values, the whiskers represent the non-outlier range, and the asterisks and open squares indicate outliers and extremes, respectively. A linear fit for the medians was plotted to show the trend of change. Differences across aspect classes were evaluated using Kruskal-Wallis H (KW-H) test. Aspect class: 1: shady aspect; 2: semi-shady aspect; 3: semi-sunny aspect; 4: sunny aspect.

Energy distribution in a warming trend on slopes of different aspects had contrasting effects on woody plant structural diversity (Figs 5,6,7 and 8). From a shady slope (cold slope) to a warmer slope, except for adult trees, seedlings, saplings, and individuals of all sizes combined decreased significantly in number of stems (Kruskal-Wallis test, *P* < 0.0001, Fig. 5), number of species (Kruskal-Wallis test, *P* < 0.001, Fig. 6), and the number of stems of the most abundant species (*N*_max_) (Kruskal-Wallis test, *P* < 0.001, Fig. 7). Adult trees increased significantly in number of stems (Kruskal-Wallis test, *P* < 0.0001, Fig. 5b) and *N*_max_ (Kruskal-Wallis test, *P* = 0.0232, Fig. 7b), but decreased, though not significantly, in number of species (Kruskal-Wallis test, *P* = 0.5484; Fig. 6b) across a warming gradient. As for tree basal area, a surrogate variable for stand biomass and productivity, which is commonly referred to as dominance in forest community ecology, the total basal area of adult trees significantly increased (Kruskal-Wallis test, *P* < 0.0001, Fig. 8b), whereas the total basal area of saplings and seedlings significantly decreased (Kruskal-Wallis test, *P* < 0.0001, Fig. 8c,d) across a warming gradient. The performance of individuals of all sizes combined was consistent with that of adult trees in the change of total basal area from shady to sunny aspects (Kruskal-Wallis test, *P* < 0.01, Fig. 8a), because adult trees had an overwhelming weight in total basal area of all the individuals (Fig. 4b).

## Discussion

Aspect-related thermal gradient shaped forest habitats with heterogeneous energy distribution, thus driving plant structural diversity patterns. Heat load increased significantly from shady to sunny aspects. This thermal energy distribution pattern on slope aspects in a subtropical forest community is similar to those observed in tropical and temperate regions, i.e., there is a universal pattern of energy distribution along slope aspects in various regions of the northern hemisphere^7^,^11^,^25^.

Our results showed that large trees responded differently to warming as compared with small trees (saplings and seedlings). Warming favored large trees but inhibited small trees in both species diversity and productivity. The number of stems, number of species and total basal area of small trees decreased, whereas the number of stems and total basal area of large trees increased with warming. As indicated by the number of stems of the most abundant species (*N*_max_), ecological dominance of large trees increased, while that of small trees decreased across a warming gradient.

Competition for thermal energy may be the key driver for different responses to warming between large and small trees. Size-asymmetric competition is common in forest communities^26^,^27^. As for size-asymmetric competition, large trees are superior competitors for energy as compared with small trees^26^. Under size-asymmetric competition, large trees with dense canopies exclude small trees of shade-intolerant species by shading effect^28^. Increase in energy from shady to sunny aspects promotes the growth of large trees; and the increases in abundance, dominance, and tree size of large trees further enhance their competitiveness, which in turn limits the growth and dispersal of small trees and consequently leads to further decreases in the abundance, species richness, dominance, and the size of small trees^26^.

Warming may also change plant structural diversity patterns through its influence on soil characteristics. Soil temperature is usually higher on sunny and semi-sunny aspects than on semi-shady and shady aspect, whereas the soil moisture exhibits an opposite trends^7^,^29^. The mineralization of soil organic matter and soil nutrient availability are positively correlated with soil moisture^30^; therefore, the contents and availability of soil nutrients are higher on shady and semi-shady aspects than on sunny and semi-sunny aspects^31^. Furthermore, higher productivity of large trees on a warmer slope may also deplete soil fertility, which will limit seedling establishment and lower the overall species diversity.

Our results reveal that warming will increase productivity of the forest community by enhancing the growth of large trees, but reduce species diversity and inhibit the regeneration of tree seedlings and saplings at stand level. However, it appears that this finding is not compatible with the conventional species-energy hypothesis for studies of broad-scale biodiversity patterns. Studies in support of the species-energy hypothesis have indicated that plant diversity increases with increasing energy^4^,^32^; e.g., the latitudinal pattern of vegetation is manifested as increases in plant diversity with increasing heat from polar to tropical regions^6^. These contrasting conclusions likely resulted from differences in several factors, such as the scale of the studies, the range of variation in energy, and the species composition and structure of forest communities under study^2^,^6^,^33^,^34^. The water-energy dynamics hypothesis suggests that on a broad geographic scale, higher plant diversity is observed in low-latitude regions as the result of the long-term synergistic effects of energy and water regimes^6^,^35^. In contrast, at the forest stand level, with similar precipitation across subplots, thermal energy distribution is largely determined by topographic conditions. The discrepancy in broad-scale versus small-scale patterns of species diversity in relation to energy suggests that water plays a key role in structuring biodiversity patterns. The water-energy dynamics hypothesis has emphasized the covariation between water and energy as well as their coordinated effects on species diversity patterns^36^,^37^. In this sense, the decrease in woody plant species diversity in response to warming does not violate the water-energy dynamics hypothesis^36^. Furthermore, extreme climate events and changes in precipitation patterns, which are increasingly frequent under climate warming, will exacerbate the decreasing of woody plant diversity at a small or local scale^33^,^38^,^39^,^40^. Our study suggests a viable way for predicting forest plant structural diversity change under global warming based on the spatially varying heat load distribution across various slope aspects.

## Additional Information

**How to cite this article**: He, S. *et al*. Topography-associated thermal gradient predicts warming effects on woody plant structural diversity in a subtropical forest. *Sci. Rep.* **7**, 40387; doi: 10.1038/srep40387 (2017).

**Publisher's note:** Springer Nature remains neutral with regard to jurisdictional claims in published maps and institutional affiliations.

## Supplementary Material

Supplementary Table S1

## Figures and Tables

**Table 1:** Correlation and partial correlation of woody plant structural diversity attributes with heat load index, having controlled for the influence of elevation.

Attribute	Heat load index	
	r	Partial r
Number of stems		
All sizes	−0.34***	−0.28***
Adult	0.15*	0.28***
Sapling	−0.32***	−0.27***
Seedling	−0.31***	−0.29***
Number of species		
All sizes	−0.29***	−0.35***
Adult	−0.11	−0.32***
Sapling	−0.25***	−0.32***
Seedling	−0.31***	−0.29***
Number of stems of the most abundant species		
All sizes	−0.30***	−0.17**
Adult	0.09	0.35***
Sapling	−0.33***	−0.24***
Seedling	−0.21**	−0.19**
Basal area		
All sizes	0.08	0.24***
Adult	0.23***	0.35***
Sapling	−0.30***	−0.26***
Seedling	−0.31***	−0.29***

All the variables were log_10_-transformed before analyses. *P < 0.05; **P < 0.01; ***P < 0.001.
