# Supplementary material for: Topography-associated thermal gradient predicts warming effects on woody plant structural diversity in a subtropical forest
Source: Sci Rep. 2017 Jan 9;7:40387. doi: 10.1038/srep40387 (PMC5220297; doi:10.1038/srep40387)
Supplement: Supplementary Table S1 [file srep40387-s1.pdf]

# Topography-associated thermal gradient predicts warming effects on woody plant structural diversity in a subtropical forest

Siyan He[+], Yonglin Zhong[+], Yudan Sun, Zhiyao Su*, Xiaorong Jia, Yanqiu Hu, Qing Zhou

College of Forestry and Landscape Architecture, South China Agricultural University, Guangzhou 510642, China

[+]These authors contributed equally to this work
*Corresponding author: zysu@scau.edu.cn

## Supplementary information

Supplementary Table S1

**Supplementary Table S1. List of tree species ≥ 1 cm DBH from the Kanghe large plot for the study.** Two out of the 250 subplots surveyed, i.e., subplots 33 and 250, were excluded from analysis due to substantial outcrops of rugged granite there. Only 27 and 10 individuals from five and two species, which are not unique to the whole plot, occurred in subplots 33 and 250, respectively. Importance value (%) = (Relative abundance + Relative frequency + Relative basal area)/3.

| Species | Abundance | Frequency | Basal area ($m^2$) | Importance value (%) |
|---|---|---|---|---|
| *Castanopsis carlesii* | 9942 | 232 | 188.33 | 25.05 |
| *Schima superba* | 6642 | 237 | 51.33 | 10.37 |
| *Ardisia quinquegona* | 4921 | 183 | 1.20 | 4.36 |
| *Itea chinensis* | 3123 | 221 | 2.85 | 3.51 |
| *Aidia pycnantha* | 1794 | 83 | 2.10 | 1.83 |
| *Litsea rotundifolia* var. *oblongifolia* | 1513 | 203 | 0.64 | 2.14 |
| *Aidia canthioides* | 1467 | 120 | 1.06 | 1.71 |
| *Engelhardtia roxburghiana* | 1242 | 163 | 10.20 | 2.62 |
| *Castanopsis fargesii* | 1124 | 191 | 28.04 | 4.32 |
| *Beilschmiedia tsangii* | 985 | 156 | 1.99 | 1.67 |
| *Elaeocarpus chinensis* | 830 | 174 | 1.25 | 1.59 |
| *Cratoxylum ligustrinum* | 806 | 42 | 0.81 | 0.83 |
| *Pterospermum heterophyllum* | 805 | 81 | 7.99 | 1.7 |
| *Ilex pubescens* | 778 | 166 | 0.17 | 1.42 |
| *Machilus velutina* | 775 | 155 | 0.23 | 1.36 |
| *Photinia prunifolia* | 763 | 148 | 1.29 | 1.41 |
| *Cunninghamia lanceolata* | 747 | 113 | 6.16 | 1.66 |
| *Camellia oleifera* | 731 | 77 | 0.29 | 0.92 |
| *Machilus chinensis* | 674 | 186 | 4.07 | 1.81 |
| *Cinnamomum porrectum* | 667 | 179 | 6.36 | 1.98 |
| *Eurya macartneyi* | 635 | 166 | 0.37 | 1.34 |
| *Diospyros morrisiana* | 633 | 179 | 0.92 | 1.46 |
| *Adinandra millittii* | 571 | 108 | 0.40 | 0.99 |
| *Castanopsis hystrix* | 516 | 117 | 9.62 | 1.84 |
| *Schefflera octophylla* | 504 | 157 | 2.88 | 1.43 |
| *Styrax odoratissima* | 488 | 139 | 0.78 | 1.13 |
| *Styrax suberifolia* | 481 | 93 | 2.65 | 1.06 |
| *Loropetalum chinensis* | 406 | 15 | 0.20 | 0.37 |
| *Eurya brevistyla* | 333 | 112 | 0.23 | 0.84 |
| *Sapium discolor* | 301 | 108 | 3.86 | 1.13 |
| *Tarenna mollissima* | 299 | 99 | 0.05 | 0.73 |
| *Cryptocarya chinensis* | 292 | 99 | 0.77 | 0.79 |
| *Ficus variolosa* | 287 | 119 | 0.24 | 0.84 |
| *Casearia villilimba* | 273 | 93 | 0.41 | 0.71 |

| Species | Abundance | Frequency | Basal area ($m^2$) | Importance value (%) |
|---|---|---|---|---|
| *Choerospondias axillaris* | 212 | 90 | 8.95 | 1.44 |
| *Olea dioica* | 205 | 50 | 0.32 | 0.43 |
| *Daphniphyllum oldhamii* | 201 | 77 | 0.81 | 0.62 |
| *Euonymus laxiflorus* | 133 | 31 | 0.02 | 0.26 |
| *Garcinia multiflora* | 115 | 48 | 0.18 | 0.35 |
| *Adina pilulifera* | 114 | 41 | 0.05 | 0.3 |
| *Reevesia thyrsoidea* | 112 | 29 | 0.64 | 0.29 |
| *Glochidion eriocarpum* | 106 | 43 | 0.05 | 0.3 |
| *Elaeocarpus sylvestris* | 100 | 58 | 0.39 | 0.41 |
| *Liquidambar formosana* | 97 | 28 | 1.43 | 0.34 |
| *Toxicodendron succedaneum* | 97 | 45 | 0.14 | 0.32 |
| *Evodia lepta* | 95 | 43 | 0.04 | 0.3 |
| *Ixonanthes chinensis* | 84 | 25 | 0.26 | 0.21 |
| *Homalium cochinchinense* | 82 | 39 | 0.03 | 0.26 |
| *Raphiolepis indica* | 76 | 44 | 0.01 | 0.29 |
| *Symplocos lancifolia* | 75 | 36 | 0.11 | 0.25 |
| *Symplocos congesta* | 69 | 21 | 0.06 | 0.16 |
| *Alangium chinense* | 67 | 39 | 1.57 | 0.4 |
| *Bridelia fordii* | 66 | 27 | 0.31 | 0.22 |
| *Camellia salicifolia* | 62 | 13 | 0.02 | 0.11 |
| *Litsea machiloides* | 61 | 18 | 0.04 | 0.14 |
| *Diospyros kaki* | 60 | 23 | 0.24 | 0.18 |
| *Vitex quinata* | 57 | 31 | 0.25 | 0.23 |
| *Gardenia jasminoides* | 56 | 41 | 0.01 | 0.26 |
| *Syzygium grijsii* | 56 | 21 | 0.01 | 0.15 |
| *Pinus massoniana* | 53 | 21 | 2.29 | 0.36 |
| *Rhodomyrtus tomentosa* | 53 | 12 | 0.02 | 0.1 |
| *Eurya acuminata* | 51 | 23 | 0.03 | 0.16 |
| *Pyrus calleryana* | 50 | 15 | 0.14 | 0.13 |
| *Antidesma japonicum* | 45 | 22 | 0.01 | 0.15 |
| *Pithecellobium clypearia* | 43 | 26 | 0.62 | 0.22 |
| *Myrica rubra* | 42 | 21 | 0.37 | 0.17 |
| *Ficus superba* | 39 | 11 | 0.36 | 0.12 |
| *Strophanthus divaricatus* | 39 | 20 | 0.01 | 0.13 |
| *Vernicia montana* | 38 | 23 | 1.11 | 0.25 |
| *Psychotria rubra* | 37 | 17 | 0.01 | 0.12 |
| *Litsea cubeba.* | 35 | 13 | 0.03 | 0.1 |
| *Lindera communis* | 33 | 15 | 0.40 | 0.14 |
| *Syzygium buxifolium* | 31 | 20 | 0.02 | 0.13 |
| *Eurya distichophylla* | 29 | 14 | 0.01 | 0.09 |
| *Celtis biondii* | 27 | 10 | 0.16 | 0.09 |

| Species | Abundance | Frequency | Basal area ($m^2$) | Importance value (%) |
|---|---|---|---|---|
| *Ehretia longiflora* | 27 | 20 | 0.06 | 0.13 |
| *Ardisia elegans* | 26 | 16 | 0.01 | 0.1 |
| *Artocarpus hypargyraeus* | 26 | 9 | 0.15 | 0.08 |
| *Daphniphyllum calycinum* | 26 | 15 | 0.04 | 0.1 |
| *Diplospora dubia* | 25 | 17 | 0.01 | 0.11 |
| *Ficus fistulosa* | 25 | 8 | 0.04 | 0.06 |
| *Celtis vandervoetiana* | 23 | 11 | 0.27 | 0.1 |
| *Vitex negundo* | 22 | 10 | 0.01 | 0.07 |
| *Canarium album* | 21 | 3 | 0.60 | 0.09 |
| *Castanopsis eyrei* | 20 | 8 | 0.61 | 0.11 |
| *Mallotus philippinensis* | 20 | 15 | 0.04 | 0.1 |
| *Ormosia semicastrata* | 20 | 5 | 0.02 | 0.04 |
| *Viburnum fordiae* | 20 | 4 | 0.03 | 0.04 |
| *Ormosia indurata* | 18 | 4 | 0.02 | 0.04 |
| *Blastus cochinchinensis* | 17 | 3 | 0.00 | 0.03 |
| *Turpinia arguta* | 15 | 9 | 0.01 | 0.06 |
| *Pygeum henryi* | 13 | 8 | 0.15 | 0.06 |
| *Ficus hirta* | 11 | 9 | 0.00 | 0.06 |
| *Photinia beauverdiana* | 11 | 7 | 0.10 | 0.05 |
| *Pithecellobium lucidum* | 11 | 8 | 0.01 | 0.05 |
| *Garcinia oblongifolia* | 10 | 7 | 0.01 | 0.04 |
| *Alchornea trewioides* | 9 | 4 | 0.00 | 0.03 |
| *Alniphyllum fortunei* | 9 | 6 | 0.04 | 0.04 |
| *Raphiolepis salicifolia* | 9 | 7 | 0.00 | 0.04 |
| *Wikstroemia indica* | 9 | 8 | 0.00 | 0.05 |
| *Ilex kwangtungensis* | 8 | 4 | 0.00 | 0.03 |
| *Clerodendrum fortunatum* | 7 | 4 | 0.00 | 0.03 |
| *Zanthoxylum myriacanthum* | 7 | 4 | 0.02 | 0.03 |
| *Callicarpa kochiana* | 6 | 5 | 0.00 | 0.03 |
| *Paulownia fortunei* | 6 | 4 | 0.55 | 0.08 |
| *Stachyurus chinensis* | 6 | 4 | 0.00 | 0.03 |
| *Dalbergia balansae* | 5 | 4 | 0.08 | 0.03 |
| *Styrax tonkinensis* | 5 | 3 | 0.02 | 0.02 |
| *Cerasus campanulata* | 4 | 4 | 0.01 | 0.02 |
| *Maesa perlarius* | 4 | 3 | 0.00 | 0.02 |
| *Rhus chinensis* | 4 | 1 | 0.00 | 0.01 |
| *Syzygium rehderianum* | 4 | 2 | 0.00 | 0.01 |
| *Albizia kalkora* | 3 | 3 | 0.01 | 0.02 |
| *Celtis tetrandra* ssp. *sinensis* | 3 | 2 | 0.01 | 0.01 |
| *Diospyros eriantha* | 3 | 3 | 0.00 | 0.02 |
| *Gleditsia fera* | 3 | 1 | 0.34 | 0.04 |

| Species | Abundance | Frequency | Basal area ($m^2$) | Importance value (%) |
|---|---|---|---|---|
| *Ilex triflora* | 3 | 3 | 0.00 | 0.02 |
| *Rhododendron farrerae* | 3 | 1 | 0.00 | 0.01 |
| *Tutcheria championii* | 3 | 1 | 0.05 | 0.01 |
| *Adenanthera pavonina* | 2 | 2 | 0.00 | 0.01 |
| *Albizia macrophylla* | 2 | 1 | 0.01 | 0.01 |
| *Ardisia crenata* | 2 | 2 | 0.00 | 0.01 |
| *Endospermum chinense* | 2 | 1 | 0.00 | 0.01 |
| *Eurya hebeclados* | 2 | 1 | 0.00 | 0.01 |
| *Evodia meliaefolia* | 2 | 1 | 0.09 | 0.01 |
| *Ficus beecheyana* | 2 | 2 | 0.00 | 0.01 |
| *Glochidion wrightii* | 2 | 2 | 0.00 | 0.01 |
| *Ilex rotunda* | 2 | 1 | 0.01 | 0.01 |
| *Machilus breviflora* | 2 | 2 | 0.02 | 0.01 |
| *Machilus pauhoi* | 2 | 1 | 0.01 | 0.01 |
| *Melastoma candidum* | 2 | 1 | 0.00 | 0.01 |
| *Nyssa sinensis* | 2 | 2 | 0.02 | 0.01 |
| *Pyrus pyrifolia* | 2 | 1 | 0.02 | 0.01 |
| *Sapindus saponaria* | 2 | 2 | 0.05 | 0.02 |
| *Sorbus caloneura* | 2 | 2 | 0.05 | 0.02 |
| *Sterculia lanceolata* | 2 | 1 | 0.03 | 0.01 |
| *Alangium kurzii* | 1 | 1 | 0.00 | 0.01 |
| *Alleizettella leucocarpa* | 1 | 1 | 0.02 | 0.01 |
| *Crataegus cuneata* | 1 | 1 | 0.06 | 0.01 |
| *Daphniphyllum macropodum* | 1 | 1 | 0.01 | 0.01 |
| *Elaeocarpus japonicus* | 1 | 1 | 0.00 | 0.01 |
| *Euonymus nitidus* | 1 | 1 | 0.02 | 0.01 |
| *Eurya japonica* | 1 | 1 | 0.00 | 0.01 |
| *Ficus formosana* | 1 | 1 | 0.00 | 0.01 |
| *Ficus pandurata* | 1 | 1 | 0.02 | 0.01 |
| *Hovenia acerba* | 1 | 1 | 0.01 | 0.01 |
| *Ilex memecylifolia* | 1 | 1 | 0.00 | 0.01 |
| *Ilex viridis* | 1 | 1 | 0.00 | 0.01 |
| *Melastoma affine* | 1 | 1 | 0.00 | 0.01 |
| *Pinus elliottii* | 1 | 1 | 0.07 | 0.01 |
| *Sarcosperma laurinum* | 1 | 1 | 0.00 | 0.01 |
| *Syzygium hancei* | 1 | 1 | 0.00 | 0.01 |
| *Wikstroemia canescens* | 1 | 1 | 0.00 | 0.01 |
